# On the Effects of Mechanical Stress of Biological Membranes in Modeling of Swelling Dynamics of Biological Systems

**DOI:** 10.1038/s41598-020-65217-4

**Published:** 2020-05-21

**Authors:** Igor Khmelinskii, Vladimir I. Makarov

**Affiliations:** 1 Faculty of Science and Technology, Department of Chemistry and Pharmacy, and Center of Electronics, Optoelectronics, and Telecommunications, University of Algarve, Faro, Portugal; 2 Department of Physics, University of Puerto Rico, Rio Piedras Campus, San Juan, USA

**Keywords:** Computational models, Biomaterials - cells

## Abstract

We highlight mechanical stretching and bending of membranes and the importance of membrane deformations in the analysis of swelling dynamics of biological systems, including cells and subcellular organelles. Membrane deformation upon swelling generates tensile stress and internal pressure, contributing to volume changes in biological systems. Therefore, in addition to physical (internal/external) and chemical factors, mechanical properties of the membranes should be considered in modeling analysis of cellular swelling. Here we describe an approach that considers mechanical properties of the membranes in the analysis of swelling dynamics of biological systems. This approach includes membrane bending and stretching deformations into the model, producing a more realistic description of swelling. We also discuss the effects of membrane stretching on swelling dynamics. We report that additional pressure generated by membrane bending is negligible, compared to pressures generated by membrane stretching, when both membrane surface area and volume are variable parameters. Note that bending deformations are reversible, while stretching deformation may be irreversible, leading to membrane disruption when they exceed a certain threshold level. Therefore, bending deformations need only be considered in reversible physiological swelling, whereas stretching deformations should also be considered in pathological irreversible swelling. Thus, the currently proposed approach may be used to develop a detailed biophysical model describing the transition from physiological to pathological swelling mode.

## Introduction

Electrodiffusion and swelling operate in a wide variety of biological systems^1–6^. These processes are important in mammalian cells and subcellular organelles such as mitochondria, nuclei and liposomes, where ion transport takes place through specialized membranes. Biophysical models of electrodiffusion with or without swelling have been presented and extensively analyzed^7–13^. However, the role of membrane stretching has been largely ignored in previous studies. Recently developed models^14–20^ included membrane elasticity and the mechanisms of ion diffusion/electrodiffusion to the membrane surface keeping the electrolyte balance between cell cytoplasm and intercellular fluid. The theoretical models developed earlier^14–20^ were based on the Euler-Lagrange equation describing mechanical tension of the bent membrane, i.e. the only mechanical property addressed was bending tension developed of the deformed membrane. Thus, considering a bilayer phospholipid membrane, its elasticity was characterized by a set of bending tensile parameters. The previous theoretical approaches^14–20^ considered dynamics of the *S/V* evolution, where *S* is the membrane surface area and *V* is the volume enclosed by the membrane, and *S* was constant during swelling. However, the effects of stretching deformations (with *S* being a variable parameter) have not been considered before. Thus, this theory should be upgraded to take into consideration the membrane stretching stress created by swelling. However, presently it is not possible to use the classical membrane elasticity theory together with the model of biophysical processes going on in the system, due to mathematical complexities inherent to the resulting description with a set of partial differential equations^21,22^. Therefore, the proposed theoretical approach has only a limited application in the more complex models capable of simulating swelling dynamics of real systems. Note that stretching stress may induce structural changes in the membrane. As it was shown earlier^23^, transition pores are created in a vesicular membrane by stretching. These transition pores are an important factor controlling swelling dynamics, and permeability transition pore (PTP) dynamics was studied in mitochondria^24,28^. However, the mechanism of PTP opening in mitochondria is poorly understood on molecular level.

Several computational models describing changes in the mitochondrial volume have been developed, reproducing swelling dynamics of these organelles^26–28^. Recently, we have proposed a simplified mathematical approach to the analysis of mitochondrial swelling dynamics, which includes stretching mechanical properties of the inner mitochondrial membrane^24,25^. This approach allowed to describe transition from the reversible (physiological) to the irreversible (pathological) mitochondrial swelling mode.

Presently we report a simple approach incorporating membrane mechanical properties into the description of swelling dynamics of biological systems in both reversible and irreversible modes. Our current focus was on the tools describing the membrane mechanical properties, and on the role of these properties in swelling dynamics. To facilitate the analysis of this problem, we simulated swelling dynamics of a closed membrane (modeling e.g. erythrocytes), with an emphasis on the initial Na^+^ and Cl^−^ ionic concentrations inside/outside the cell, and water transport across the membrane. The cell was initially shaped as an oblate axisymmetric ellipsoid. Currently we present the resulting biophysical model, as an example of using the membrane-property tools for a simplified biological system. These tools should be useful in the evaluation of biophysical models of different levels of complexity and in the study of volume changes of biological structures. We also evaluated the contribution of these tools to the models of equilibrium (quasistationary) conditions, comparing their results with those obtained by the usage of the classical biomembrane theory^17–20^, and discussing the relations between the key parameters of the two theoretical approaches.

## Model description

**Figure 1:**
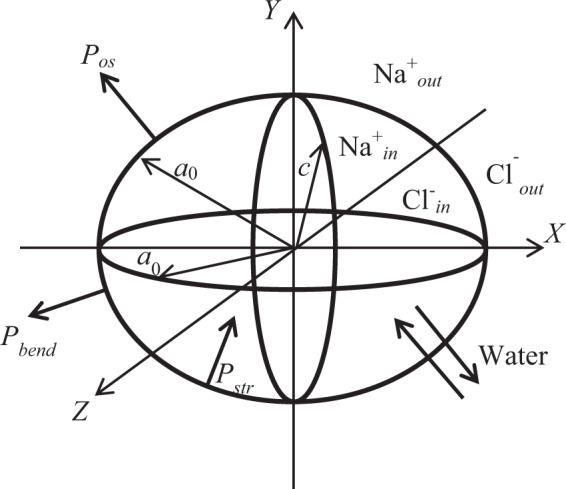
Erythrocyte modelled by an oblate axisymmetric ellipsoid; *a* = *b* = *a*_0_ = 8 µm and *c* = 2 µm.

We tested our theoretical approach on a model of closed membrane that describes an erythrocyte. An erythrocyte is approximately a disk 6–8 μm in diameter and 2–3 μm thick^29^. For simplicity, we modeled an erythrocyte by an oblate axisymmetric ellipsoid, shown in Fig. 1.

In equilibrium initial conditions, the ellipsoid geometrical parameters were *a* = *b* = *a*_0_ = 8 μm and *c* = 2 μm, with the ellipsoid axes coinciding with the respective *x, y* and *z* axes of the Cartesian referential. The rigidity parameters describing the mechanical properties of the membrane will be introduced below. Note that classical membrane elasticity theory developed earlier^17–20^ is mainly applied to the analysis of biomembrane shape (*S/V* is variable with *S* = constant), when stretching stress may be neglected in a direct problem. However, presently we are considering an inverse problem, where the membrane shape is given, stretching stress is significant, and for the given shape, bending rigidity parameters and pressure induced by the bending tension should be deduced.

### Classical elastic membrane model

To simplify the analysis of membrane bending tension, we used the classical theory of elastic membranes developed earlier^30,31^ for phospholipid bilayer membranes, with the free energy of the membrane given by:

1 $$\begin{array}{c}F=\mathop{\int }\limits_{S}({f}_{c}+\lambda )dA+\mathop{\int }\limits_{V}\Delta pdV\\ {f}_{c}=\frac{{k}_{c}}{2}{(H+{c}_{0})}^{2}+{k}_{d}K\end{array}$$

here, *H* and *K* are the respective mean and Gaussian curvatures, which were determined and calculated using the procedure developed earlier^32,33^, *dA* and *dV* are the area and volume elements, of the membrane surface, and of the space enclosed by the membrane, respectively. Also, *k*_*c*_ and *k*_*d*_ are the bending modulus values, *c*_0_ is the spontaneous curvature of the membrane, which was neglected in our analysis due to planar spontaneous membrane structure, *λ* is the Lagrangian multiplier and Δ*p* is the additional pressure inside the membrane, caused by bending tension. Note that phospholipid bilayer model for the membrane was only used to calculate the bending-induced pressure. The Euler-Lagrange equation corresponding to this energy functional for a spherical vesicle is given by^34^:

2 $$\Delta p-2\lambda H+{k}_{c}(2H+{c}_{0})(2{H}^{2}-{c}_{0}H-2K)+2{k}_{c}({\nabla }^{2}H)=0$$

The equations for the energy functional of an oblate axisymmetric erythrocyte are quite complex and may only be analyzed numerically. These equations have four unknown parameters: *k*_*c*_, *k*_*d*_, Δ*p*, and *λ*, where Δ*p* and *λ* depend on the membrane shape. Note that presently we limit ourselves to the analysis of a simple biomembrane elasticity theory, which neglects all of the effects of the respective cytoskeleton^30,31^. Our objective was thus to calculate the values of the Δ*p*, *k*_*c*_ and *k*_*d*_ parameters at different levels of cell swelling.

### Stretching stress in a biomembrane

As we already noted, our model neglects the transport of neutral or ionic species through the membrane and the resulting concentration difference of Na^+^ and Cl^−^ across the membrane is therefore controlled by water transport only. Thus, here we focus on the analysis of tools that describe membrane stretching.

Osmotic pressure may be described by the following relationship:^35^

3 $$\Delta {P}_{os}={k}_{B}TN\sum _{i}({C}_{i}^{in}-{C}_{out}^{in})$$

where *k*_*B*_ is the Boltzmann constant, *N* is the Avogadro number, *T* is the absolute temperature, *C*_*i*_^*in*^ and *C*_*i*_^*out*^ are the concentrations of the *i-th* species at the two sides of the membrane. In a biological system, osmotic pressure induces cell swelling through the influx of water. The membrane properties defining its mechanical stress due to swelling may be described by a second-rank rigidity tensor, in a biological object with an arbitrary 3D configuration^24,25^.

For simplicity, we shall model erythrocytes by oblate ellipsoids (see Fig. 1). Figure 1 also shows a referential that is rigidly connected to the biological system, with its Cartesian *x-, y-*, and *z*-axes directed respectively along with the *a, b*, and *c* axes of the oblate ellipsoid. The rigidity tensor becomes diagonal in this referential^24,25^, with only the diagonal components *g’*_*ii*_ (*i* = *x, y, z*) different from zero. Note that these tensor components are directly dependent on the membrane properties including its biochemical composition and structure, its permeability with respect to transport of different ionic and neutral species, its mechanical stability, and its physicochemical activity. Besides, there are external parameters (concentration gradient of ions/neutral species, protein concentrations, temperature, metabolic activity, etc.) that are strongly coupled to the internal membrane parameters. For an axisymmetric oblate ellipsoid (*a* = *b*), we obtain *g’*_*xx*_ = *g’*_*yy*_^24,25^. Using the material resistance theory^36^, diagonal tensor components may be presented as:

4 $$\begin{array}{c}g{{\prime} }_{xx}=g{{\prime} }_{yy}={g}_{0}={g}_{00}\left(1-\frac{{\beta }_{0}\Delta {r}^{{n}_{1}}}{1+{\beta }_{0}\Delta {r}^{{n}_{1}}}\right)\\ {g}_{zz}={g}_{zz,0}\left(1-\frac{{\beta }_{z}\Delta {z}^{{n}_{1}}}{1+{\beta }_{z}\Delta {z}^{{n}_{1}}}\right)\end{array}$$

These expressions of the rigidity tensor components include membrane disruption at large mechanical deformations (irreversible pathological swelling), where *β*_*i*_ (*i* = 0 and *z*) and *n*_1_ are the respective phenomenological parameters, introduced to describe the reduction in stress at large deformations^24,25^. The ellipsoid deformations are Δ*r* along the *x* and *y* axes, and Δ*z* along the *z* axis, respectively. Here *g*_00_ and *g*_*zz*,0_ are the membrane rigidity parameters describing the membrane properties in the *x, y* and *z* directions, respectively, at infinitely small deformations. According to Eq. (4), the membrane rigidity approaches zero asymptotically at large mechanical deformations, to describe irreversible pathological swelling and cell membrane mechanical disruption. The parameter values were determined by fitting the available experimental data with biophysical models of different complexity levels, where swelling dynamics was described taking into account the membrane mechanical properties^23,25^. Note that we used phenomenological rigidity tensor parameters, dependent on membrane composition and structure. These rigidity parameters may be affected e.g. by viscous elements present in the membrane. Note that the rigidity tensor formalism was already used to describe swelling dynamics of biological objects^37,38^. However, presently we produced an improved version of this formalism. As we already noted, membrane rigidity should depend on membrane composition and structure, and will be explored in a follow-up study.

The internal pressure induced by membrane stretching stress may be represented as follows:^24,25^

5 $$\Delta {P}_{IMM}(t)=\frac{2{g}_{0}(t)\Delta r(t)+{g}_{zz}(t)\Delta z(t)}{S(t)}$$

where *S*(*t*) is the time-dependent surface area of the membrane, *g*_0_(*t*) and *g*_*zz*_(*t*) are the rigidity tensor components represented by Eq. (4), which are time-dependent due to the time dependence of the Δ*r*(*t*) and Δ*z*(*t*) deformations. The surface area and volume of an axisymmetric oblate ellipsoid were given by Tikhonov and Samarskii^39^, and we have discussed the respective calculations earlier^24,25^.

Swelling dynamics is then described by the equation:

6 $$\frac{dV}{dt}=S(t){p}_{w}(t)(\Delta {P}_{os}-\Delta {P}_{IMM})$$

Here, *p*_*w*_(*t*) is the time-dependent membrane permeability for water. The changes in the internal concentrations of the *i-th* ionic or neutral species due to swelling were described by:

7 $$\frac{d{C}_{i}^{in}}{dt}=-\frac{{C}_{i}^{in}}{V(t)}\frac{dV(t)}{dt}$$

Equations (6, 7) provide a correct description of swelling by taking into account the mechanical properties of the biological membrane, primarily as regards stretching stress. Similar equations combined with a comprehensive description of the respective biophysical processes may be used to analyze the swelling of cells or cellular organelles such as mitochondria^24,25^. Other membrane-specific biophysical processes may include the generation of concentration gradients of ionic and neutral species and transport of these species through the membrane. The usage of such combined models to predict and model the experimental data could generate quantitative estimates for the rigidity tensor components, as has already been done for the inner mitochondrial membrane^24,25^.

### Numerical simulations

#### Numerical simulations of a closed membrane (erythrocyte) surface area and volume dynamics using the theory of subsection 2.2

Since the parameter values describing the rigidity tensor components, Eq.(4), are unknown, we used the values of the respective parameters estimated earlier^24,25^ for the inner mitochondrial membrane isolated from murine heart. The values of these parameters are listed in Table 1. Note that this selection of the parameter values is quite reasonable, because the protein-to-lipid ratio equals 80:20 in the inner mitochondrial membrane (IMM), and 50:50 in the outer membrane^40^. The IMM rigidity is determined by the amount of cholesterol in the IMM, which depends on the type of cells used as the mitochondrial source^41^.

**Table 1:** The membrane rigidity tensor components^24,25^.

Parameter	Value	Units
*n*_1_	4	–
*g*_*zz*,0_	0.008 ± 0.001	dyn/nm
*g*_00_	0.0101 ± 0.0011	dyn/nm
*β*_*z*_	(1.6 ± 0.2)×10^4^	μm^–*n*1^
*β*_0_	(1.8 ± 0.2)×10^4^	μm^–*n*1^
*p*_*w*_	(3.1 ± 0.7)×10^2^	μl·min^–1^Pa^–1^μm^–2^

The values of the tensor components were exchanged as compared to our previous publications^23,25^, since a prolate axisymmetric ellipsoid was considered earlier, while currently we used an oblate axisymmetric ellipsoid.

**Figure 2:**
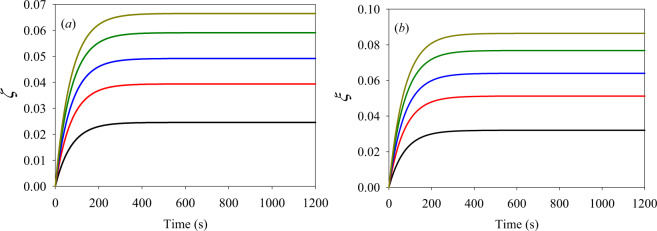
Dynamics of the (**a**) *ζ* and (**b**) *ξ* parameters induced by the cell swelling, bottom to top: 10 mM NaCl – black line; 20 mM NaCl – red line; 30 mM NaCl – blue line; 40 mM NaCl – green line and 50 mM NaCl – yellow line.

All of the simulations were carried out in a system, where the cell volume was much smaller than that of the surrounding medium, equivalent to assuming constant external concentration of Na^+^ and Cl^−^, equal to 1 mM in all of the numerical experiments. However, the initial internal concentrations of the same ions varied from 10 mM to 50 mM, and the dynamics of the cell surface area and volume were calculated for each of the sets of the initial concentrations. The results of calculations are presented as dimensionless parameters:

8 $$\zeta (t)={a}_{0}(t)/{a}_{0}(0)-1,\,{\rm{a}}{\rm{n}}{\rm{d}}\,\,\xi (t)=c(t)/c(0)-1,$$

describing relative changes in the lengths of the two axes of the ellipsoid. Numerical analysis was carried out using homemade FORTRAN code, and the results are shown in Fig. 2.

We observe saturation effects for the inner concentrations of the Na^+^ and Cl^−^ ions in all cases. The saturation effects originate in the equality of the water fluxes going in and out of the cell. The incoming water flux is generated by the osmotic pressure, causing a dilution of ionic concentrations due to the growing cellular volume, while the outgoing water flux is generated by the internal cell pressure caused by the membrane stretching. Presently we did not analyze the transition conditions from the reversible to the irreversible swelling, as our model system is insufficiently comprehensive to appropriately describe a real erythrocyte. As we noted above, our main objective was to draw attention to the importance of the mechanical stretching of the membrane in its swelling behavior. Therefore, here we considered a simple model simulating the shape of an erythrocyte. In future, a more detailed biophysical model including exchange of additional ionic and neutral solutes in an erythrocyte and mechanical properties of its membrane will be considered in swelling modeling.

We have to note that curves in Fig. 2a have very similar rise time. This result follows directly from Eq. (6), which in an approximate form may be presented as follows:

9 $$\begin{array}{c}\frac{d\Delta V}{dt}=\bar{S}{\bar{p}}_{w}(\Delta {P}_{os}-\alpha \Delta V)\\ \Delta {P}_{IMM}\approx \alpha \Delta V\end{array}$$

where $\bar{S}$ and ${\bar{p}}_{w}$ are the average surface area and water permeability values and *α* is the coefficient coupling the pressure induced by mechanical stretching and volume changes. The solution of Eq.(9) may be presented as follows:

10 $$\Delta V(t)=\frac{\Delta {P}_{os}}{\alpha }(1-{e}^{-\alpha \bar{S}{\bar{p}}_{w}t})$$

**Figure 3:**
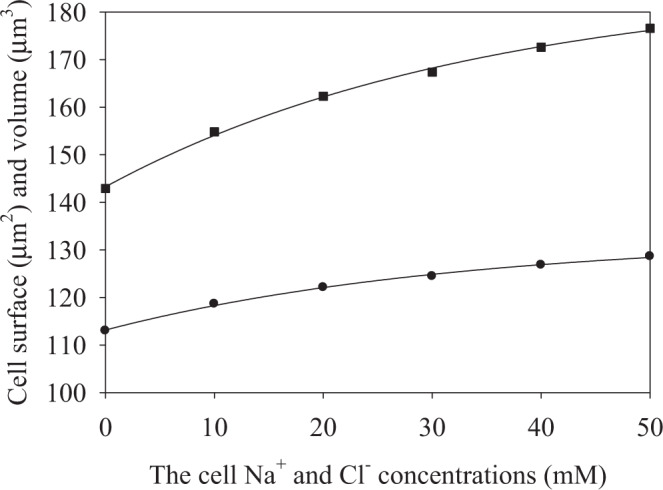
The saturated values of the cell surface area (circles) and volume (squares) versus the initial intracellular Na^+^ and Cl^–^ concentrations.

Equation (10) shows that the risetime is $\alpha \bar{S}{\bar{p}}_{w}$, and it is independent on the osmotic pressure, while the saturation value is dependent on the osmotic pressure. The same is valid for the data of Fig. 2b. Using the ellipsoid parameter dynamics shown in Fig. 2, we calculated the saturation values of the cell surface area and volume, keeping ellipsoidal shape at all values of the internal ionic concentrations during swelling. The calculated saturation values of the surface area and volume are shown in Fig. 3.

Note the nonlinearity of the plots, which should be due to dilution of the solutes and the linearly growing membrane stretching, thus, no significant deviations of the mechanical stress from linearity were detected in these numerical experiments. Next, we shall reanalyze the results of Fig. 3 using the classical membrane elasticity theory (Section 2.1).

### Classical elastic membrane theory used to analyze membrane stretching

The equilibrium shape of our model system was described by an oblate axisymmetric ellipsoid, with the parameter values listed in Table 2 at different solute concentrations.

**Table 2:** Equilibrium shape of an oblate axisymmetric ellipsoid modeling an erythrocyte.

[Na^+^] = [Cl^−^], mM	*a*_0_, μm	*C*, μm
0	8.00	2.00
10	8.20	2.06
20	8.32	2.10
30	8.39	2.13
40	8.47	2.15
50	8.53	2.17

Taking into account the information in Table 1 and the relationship coupling the stretching rigidity parameters with the respective bending rigidity constants for a homogeneous sheet^42^, we carried out numerical optimization of the *k*_*c*_ and *k*_*d*_ parameter values (see Table 3). Taking into account the estimated values of the bending rigidity constants and Eq. (2), we additionally calculated the pressure induced by the membrane bending stress. The numerical analysis was carried using home-made FORTRAN code, and the respective results are listed in Table 3.

**Table 3:** Optimized values of the membrane bending rigidity constants *k*_*c*_ and *k*_*d*_, and the bending contribution to pressure Δ*p*.

[Na^+^], [Cl^−^], mM	*k*_*c*_, 10^−5^ erg	*k*_*d*_, 10^−5^ erg	Δ*p*, Pa
0	1.38	0.71	11.2
10	1.40	0.73	9.6
20	1.37	0.70	8.3
30	1.36	0.76	7.4
40	1.38	0.71	6.7
50	1.34	0.72	6.0

**Figure 4:**
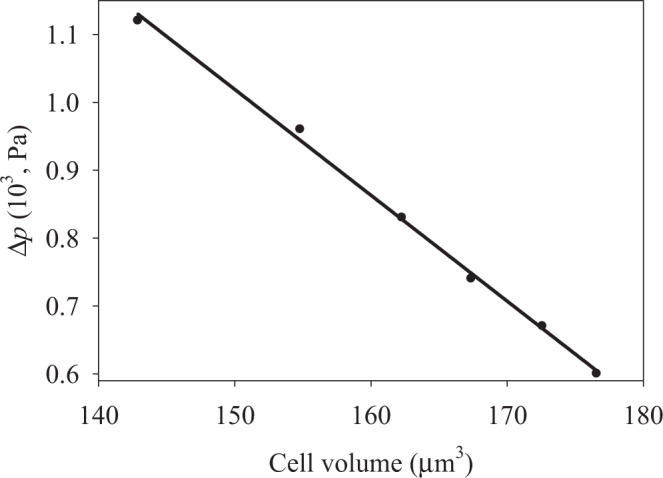
Bending-induced pressure inside the cell in function of its volume for an oblate axisymmetric ellipsoidal cell.

We see that bending rigidity is independent on the swelled cell shape within the range of the parameter values tested. The pressure Δ*p* induced by the membrane bending stress decreases with increasing volume, as shown in Fig. 4.

Note that the Δ*p* vs. *V* dependence may be fitted by a linear function, with the intercept and slope equal to 3.359 Pa and –0.0156 Pa/μm^3^, respectively. These results are in qualitative agreement with those reported earlier^19^, where it was shown that bending-induced pressure varies as *r*^–3^ for a spherical membrane. Note that bending-induced pressure is directed outside of the cell and it is perpendicular to the membrane as shown in Fig. 1, while the pressure induced by membrane stretching is directed inside the cell. In other words, bending-generated pressure attempts to restore the flat shape of the membrane, and remove the bending strain, while the stretching-induced pressure attempts to restore the equilibrium conditions. However, bending-induced pressure (Δ*p*_*bend*_ = 6 Pa at 50 mM NaCl) is typically much lower than stretching-induced pressure (Δ*p*_*str*_ = 124 Pa at 50 mM NaCl) during swelling, whereupon both *S* and *V* parameters are changed. Therefore, we may neglect bending-induced pressure in our swelling models, where reversible to irreversible swelling transition is analyzed. In the next section, we will discuss the currently developed approach to the analysis of cell swelling dynamics.

## Discussion

Most biophysical models of cell swelling reported to date have ignored the internal pressure generated in the system by membrane stretching (as we already noted, bending-induced pressure is negligible during swelling, when both *S* and *V* parameters are changing). Swelling dynamics in these models was described by the equation:^27^

11 $$\frac{dV(t)}{dt}=S(t){\eta }_{w}{k}_{B}TN\sum _{i}[{C}_{i}^{in}(t)-{C}_{out}^{in}(t)]$$

where *η*_*w*_ is the membrane permeability coefficient for water, and other parameters of Eq. (11) were defined above. The term:

12 $${J}_{w}^{in}=S(t){\eta }_{w}{k}_{B}TN\sum _{i}{C}_{i}^{in}(t)$$

describes the inflow of water, while the term:

13 $${J}_{w}^{out}=S(t){\eta }_{w}{k}_{B}TN\sum _{i}{C}_{i}^{out}(t)$$

describes the outflow of water from the cell. Assuming that the equilibrium was achieved, we have:

14 $${{J}_{w}}^{in}={{J}_{w}}^{out}$$

whereupon no more swelling will occur, corresponding to saturated swelling. As we already noted, this approach does overlook the water flux induced by the internal pressure due to membrane stretching. Therefore, Eq. (9) may produce an incorrect description of swelling. As we also noted above, dynamic effects of the internal pressure induced by bending stress were extensively studied earlier^14–20^. However, the developed model does not include the mechanisms for solute transport through the biomembrane, typically considering diffusion-limited solute transport to and from the membrane. In all of those models, as we already noted, the system volume is only changed during swelling, while the membrane surface area is kept constant, thus positive swelling dynamics corresponds to a decrease in the *S/V* ratio, while negative dynamics corresponds to growing *S*/*V* ratio at a constant value of *S*. Bending tension and pressure will be created by bending deformation of the membrane. These effects may be understood considering an elastic spherical shell filled by a liquid, which provides the minimum *S/V* ratio. If we will pump some liquid out of the shell, its shape will change, generating additional bending tension and negative internal pressure in our deformed system. Thus, the *S/V* will increase, while keeping the surface area constant. Note that bending pressure is directed outside of the shell (Fig. 1), and for a water-permeable shell, such negative pressure induces an influx of water. This effect is of opposite sign, compared to the effects generated by stretching stress of our shell.

Although the errors introduced by Eq. (9) could be negligible when the cells are incubated in physiological solution ([NaCl]_0_ ≈ 145 mM), they could become significant in cells of the renal epithelium bathed in dilute luminal fluid ([NaCl]_0_ < 50 mM)^33^. Also, Eq. (9) does not describe correctly the irreversible swelling of biological systems, which is important in pathological states of cells or subcellular organelles such as mitochondria. This problem is addressed in the present study through the analysis of the membrane stretching properties. Indeed, membrane disruption at large stretching deformations may be described using the rigidity tensor components of Eq. (4). We should consider the reversibility of membrane deformations together with its internal parameters that affect its rigidity, directly or indirectly. These include, among others, biochemical composition of the membrane, its shape, its permeability for ionic and neutral species, temperature, and biophysical/biochemical activity. The rigidity tensor parameters describing membrane stretching deformations could be evaluated experimentally by analyzing swelling dynamics based on the presently developed mathematical models. Indeed, membrane disruption should be included into the equations at large membrane strain values, when we model and analyze irreversible (pathological) swelling. The rigidity data thus obtained could be critical to understanding the membrane internal properties.

We showed above that the tools developed for the analysis of stretching deformations of biomembranes allow to describe the swelled membrane shape in equilibrium conditions, and to calculate bending rigidity parameters and bending-induced pressure in the system by applying the classical membrane elasticity theory to the equilibrium configuration. Presently we demonstrated the utility of this approach for a phospholipid bilayer membrane. However, considering a real membrane, cytoskeletal properties should be taken into account for a more correct analysis of the membrane bending tension^30^ in equilibrium shape. The tools for such an analysis were already developed earlier^14–20^, and should be combined with those discussed presently to obtain a complete description of the cellular membrane properties. We shall provide such analysis in a follow-up publication, including additionally the analysis of the irreversible cell swelling. As we already noted, the knowledge of the rigidity parameter values may help to understand the structure of biological membranes. We additionally note that both bending and stretching rigidity parameters contribute to modeling the membrane structure and properties. We also noted that bending tension effects are only important, when the *S/V* ratio is changed at constant *S*. On the other hand, stretching deformation of membrane dominates when *S* grows, and here bending stress effects may be neglected compared to those induced by stretching stress.

Thus, membrane mechanical properties should be included for the correct analysis of swelling dynamics in biological systems. Swelling caused by osmotic pressure deforms the membrane, generating a stress-dependent membrane contribution to the internal pressure in the system. This additional pressure could partially compensate for the existence of an inward osmotic gradient, leading to dissipation of water fluxes and maintenance of cell volume. The effect of membrane stress promoting the outflow of water would be independent of the activation of mechano-sensitive ion-channels that promote the exchange of osmolytes and prevent further volume increase^43–45^. Nevertheless, both mechanisms could operate to reduce the membrane tension and preserve cell function during osmotic stress. Additional studies are needed to evaluate whether the proposed model adequately simulates the experimental data on the osmotic behavior of cells. This information is critical for the contribution of the membrane mechanical properties to the osmotic behavior of cells, in particular, during alterations of extracellular tonicity (hypotonic-isotonic-hypertonic), as could be experienced by the kidney tubular cells, or during cell activation in inflammation, cell culture, and cryopreservation.

## Conclusion

In the current study, we discussed tools that describe reversible and irreversible swelling dynamics of a biomembrane induced by osmotic water influx, where membrane stretching plays a key role. It was shown that bending effects (Δ*p*_*bend*_ = 6 Pa at 50 mM NaCl) in swelling dynamics are much less important in comparison to stretchingeffects (Δ*p*_*str*_ = 124 Pa at 50 mM NaCl)... shown that bending effects (Δ*p*_*bend*_ = 6 Pa at 50 mM NaCl) in swelling dynamics are much less important in comparison to the stretching effects (Δ*p*_*str*_ = 124 Pa at 50 mM NaCl), when both volume and surface area are changed. However, bending effects should be taken into consideration, when the cell volume is a variable parameter, while keeping its surface area constant. Note that bending deformations are reversible, while stretching deformations may be irreversible, leading to membrane disruption when they exceed a certain threshold level. Therefore, bending deformations need only be considered for reversible physiological swelling, whereas stretching deformations should also be considered for pathological irreversible swelling. Thus, realistic biophysical models should include both bending and stretching deformations of biomembranes. It should be made clear that different membrane deformations are important at different levels of swelling: bending is the most important when *S/V* ≥ (*S/V*)_*min*_ at *S* constant; while stretching is the most important when *S/V* < (*S/V*)_*min*_ at both *S* and *V* variable, where (*S/V*)_*min*_ is the minimum ratio value at *S* constant.

### Limitations

The currently discussed approach includes numerous approximations, lacking dynamics of solute transport, membrane potential, intrasystem metabolism and other processes that should be all taken into consideration in real biological systems. However, presently we are developing tools that take into account membrane mechanical properties, and explore their impact on detailed biophysical models.
